# Amino-Alcohol Organic-Inorganic Hybrid Sol-Gel Materials Based on an Epoxy Bicyclic Silane: Synthesis and Characterization †

**DOI:** 10.3390/nano13172429

**Published:** 2023-08-26

**Authors:** Rui P. C. L. Sousa, Rita B. Figueira, Emanuela Callone, Sandra Dirè, Susana P. G. Costa, Maria Manuela M. Raposo

**Affiliations:** 1 Centre of Chemistry, University of Minho, Campus of Gualtar, 4710-057 Braga, Portugal; rui.sousa@quimica.uminho.pt (R.P.C.L.S.); spc@quimica.uminho.pt (S.P.G.C.); 2 “Klaus Müller” NMR Laboratory, Department of Industrial Engineering, University of Trento, Via Sommarive, 9, 38123 Trento, Italy; emanuela.callone@unitn.it (E.C.); sandra.dire@unitn.it (S.D.)

**Keywords:** organic-inorganic hybrid, sol-gel, films, synthesis, characterization

## Abstract

Organic-inorganic hybrids (OIHs) are a type of material that can be obtained using the sol-gel process and has the advantages of organic and inorganic moieties in a single material. Polyetheramines have been widely used in the preparation of this type of material, particularly in combination with epoxy-based alkoxysilanes. Nevertheless, epoxyciclohexylethyltrimethoxysilane (ECHETMS) is a promising alkoxysilane with an epoxy terminal group that is quite unexplored. In this work, four novel OIH materials were synthesized using the sol-gel method. The OIHs were based on Jeffamines^®^ of different molecular weights (D-230, D-400, ED-600, and ED-900), together with ECHETMS. The materials were characterized using multinuclear solid state NMR, FTIR, BET, UV/Vis spectroscopy, EIS, and TGA. The influence of the Jeffamine molecular weight and the suitability of these materials to act as a supporting matrix for heteroaromatic probes were assessed and discussed. The materials show interesting properties in order to be applied in a wide range of sensing applications.

## 1. Introduction

The sol-gel method is a synthetic process that has been used since the 19th century [1]. This method has been widely employed in the production of smart and multifunctional materials during the past decades [2,3,4,5]. Sol-gel materials can have very different properties due to changes in the used precursors or in the chemical conditions during the synthesis leading to a wide range of possible applications. This process is a low-temperature procedure since it requires unexpensive equipment and low energy consumption, which are interesting features from an industrial approach [6]. Besides that, it is a simple, low-cost and low environmental impact process.

Sol-gel synthesis is based on the conversion of monomers into a colloidal solution (*sol*). This solution can be shaped in the most suitable way, e.g., applied on a substrate or cast in a mold with desired dimensions, which after undergoing a chemical transformation, forms an interconnected three-dimensional network (*gel*) [6,7]. Several factors, such as pH, temperature, or gelation time, can influence the final solid material. The drying phase of the gel is the last step and depending on the temperature, the treatment allows obtaining of xerogels, aerogels, monoliths, or fibers.

Organic-inorganic hybrids (OIHs) are materials that can be obtained using this procedure [8,9,10]. These OIHs can join the features of inorganic and organic parts in a single matrix [11]. The inorganic part of OIHs is usually based on silicon or transition metal alkoxides, and organic moieties can change from simple carbon chains to more complex hydrophobic organic molecules. Moreover, the commercial diversity of available precursors allows to adjust the final chemical, mechanical, or optical properties of the matrices. When compared with other methods for the development of OIHs, the sol-gel process shows singular advantages, such as the flexibility and simplicity of the method or the low-processing temperatures [12].

Among organic precursors used in the synthesis of OIHs are polyetheramines, organic polymers bearing terminal amine groups that can react with different inorganic precursors. The reaction between a polyetheramine and an alkoxysilane can yield OIHs that can be used in the preparation of sol-gel materials, in the form of ureasilicates or aminoalcoholsilicates, in accordance with the type of bond that is established between the two precursors used. Jeffamines^®^ are commercial polyetheramines widely used in the synthesis of OIHs, with D and ED series being among the most common diamines, allowing the reaction with silanes in a 1:2 stoichiometry [13,14,15]. D series Jeffamines^®^ are generally based on a polypropylene glycol (PPG) backbone, whereas ED series are mostly based on a polyethylene glycol (PEG) backbone.

Amongst the most used alkoxysilanes for OIH preparation are 3-isocyanatepropyltriethoxysilane (3-ICPTES), 3-glycidoxypropyltrimethoxysilane (3-GPTMS), or 3-aminopropyltriethoxysilane (3-APTES). Epoxyciclohexylethyltrimethoxysilane (ECHETMS) is another interesting alkoxysilane that can be used in the synthesis of this type of material, although as far as the authors’ knowledge, there are not many materials reported based on such precursor. In fact, less than a dozen publications have been reported so far on the synthesis of OIH materials based on this precursor, with applications in the fields of coatings [16,17], photonics [18,19], and sensors [20]. Nevertheless, this precursor can bring interesting properties to the final sol-gel matrices mainly due to its cycloalkane nature, which brings some rigidity to the membrane [21]. Besides that, the epoxy ring is more reactive than in glycidoxypropyl silanes.

This work reports novel OIH material produced using the sol-gel method, based on Jeffamines^®^ D-230, D-400, ED-600, ED-900, and ECHETMS. The materials were successfully synthesized and structurally characterized using multinuclear solid-state NMR and FTIR. The dielectric properties were assessed using EIS. Thermal degradation was characterized using TGA. UV/Vis spectroscopy was performed to study the optical properties. The influence of the Jeffamine molecular weight (MW) and the suitability of these materials to act as a supporting matrix for doped chemosensors was also approached.

## 2. Materials and Methods

### 2.1. Materials

Commercial reagents [2-(3,4-epoxycyclohexyl)ethyl]trimethoxysilane (ECHETMS) (98%, Sigma-Aldrich, St. Louis, MO, USA), Jeffamines^®^ D-230, D-400, ED-600, ED-900 (Huntsman Corporation, Pamplona, Spain), and solvent acetonitrile (ACN, Fisher Scientific, Loughborough, UK) were used as received. A Millipore water purification system (Milli-Q^®^, Merck KGaA, Darmstadt, Germany) was used to obtain high-purity deionized water (resistivity > 18 MΩ cm).

### 2.2. Synthesis of OIH Matrices

In a glass container, the different Jeffamines^®^ (1 mmol) were dissolved in 500 µL of ACN. ECHETMS was then added (2 mmol—molar ratio of 1:2) and the mixture was stirred for 20 min. After addition of 100 µL of water, a homogeneous solution was obtained. Gels were then cast into Teflon molds, covered with Parafilm^®^, and kept in an oven (UNB 200, Memmert, Buechenbach, Germany) for 15 days at 40 °C. The drying step is necessary to ensure the curing of the film.

### 2.3. Characterization of OIH Matrices

#### 2.3.1. Fourier Transform Infrared Spectroscopy (FTIR)

The OIHS were characterized using FTIR on a PerkinElmer Spectrum Two instrument with ATR. The analysis was performed in the range of 500–4000 cm^−1^ with 32 scans. Xerogels were applied directly on the ATR crystal.

#### 2.3.2. Nuclear Magnetic Resonance (NMR)

The samples were characterized using solid-state NMR with a Bruker 400 WB spectrometer operating at a proton frequency of 400.13 MHz. Magic Angle Spinning (MAS) NMR spectra were obtained with single pulse or cross polarization (CP) sequence with: 100.48 MHz, contact time 2 ms, decoupling length 5.9 μs, recycle delay: 3 s, 4 k scans—for ^13^C frequency; 79.48 MHz, contact time 5 ms, decoupling length 6.3 μs, recycle delay: 10 s, 5 k scans—for ^29^Si frequency; and for single pulse π/6, 200 s and 180 scans. Samples were packed in 4 mm zirconia rotors that were spun at 6.5 kHz under air flow. Adamantane and Q_8_M_8_ were used as external secondary references. According to the common ^29^Si NMR notation, the Si species are labelled T^n^: T represents R-SiO_3_ structural units and n represents the number of bridging oxygens.

#### 2.3.3. BET

The surface area of the OIH sol-gel materials was determined using the multi-point Brunauer–Emmet–Teller method (BET, Quantachrome Autosorb AS-1) at −196 °C. Before the analysis, the samples were outgassed in a vacuum at 140 °C for 4 h.

#### 2.3.4. Electrochemical Characterization (EIS)

EIS spectra were measured in a Faraday cage, using a potentiostat/galvanostat/ZRA (Reference 600+, Gamry Instruments, Warminster, PA, USA). EIS measurements were used to characterize the resistance, electric permittivity, electrical conductivity, and capacitance of OIH xerogels. The disc films were placed between two Au electrodes (10 mm diameter and 250 μm thickness) according to the procedure already described elsewhere [22]. A 10 mV (peak-to-peak, sinusoidal) electrical potential within a frequency range from 1 × 10^6^ Hz to 0.01 Hz (10 points per decade) was applied at open circuit potential (OCP). Nyquist plots displayed the obtained data, fitted with Gamry ESA410 Data Acquisition software (v. 7.8.6).

#### 2.3.5. Thermal Characterization (TGA)

OIH xerogels were characterized using TGA on a SDT Q600 system. A temperature ramp of 15 °C min^−1^ was used between room temperature and 750 °C at a constant 100 mL min^−1^ nitrogen flux. The mass used for each sample ranged between 20 and 30 mg.

#### 2.3.6. Optical Characterization (UV/Vis Spectroscopy)

UV–Vis transmittance spectra for the new OIH films were obtained with a Shimadzu UV-2501 PC spectrophotometer, in the range of 200–800 nm.

## 3. Results and Discussion

### 3.1. Synthesis of Organic-Inorganic Hybrid (OIH) Films

**Figure 1:**
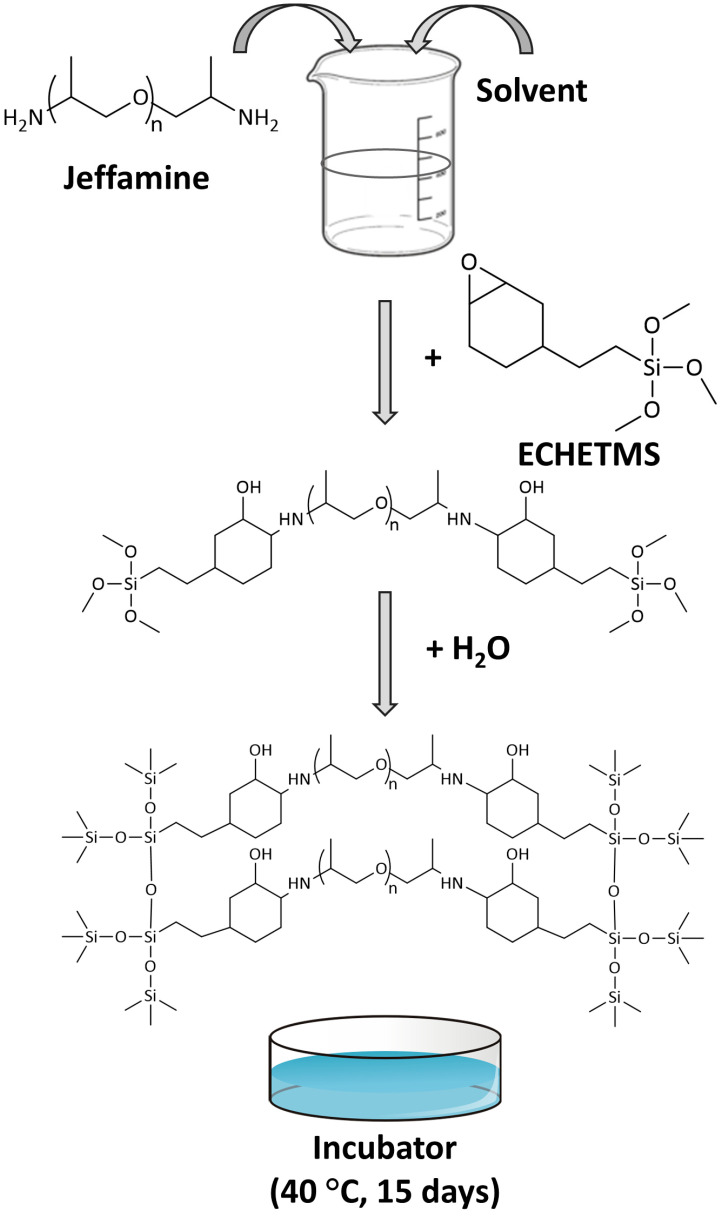
Synthesis steps of the new OIH sol-gel xerogels.

The synthesis of the novel OIH sol-gel materials started with the dissolution of the 1 mmol of the different Jeffamines in 500 µL of ACN in a glass container. ECHETMS was added in a molar ratio of Jeffamine 1:2 ECHETMS. The solution was then stirred for 20 min until a homogeneous mixture was obtained. Water was added to start the hydrolysis of the *sol* and 10 min later, the mixtures were casted into Teflon molds and kept in oven for 15 days at 40 °C. Figure 1 details the synthesis steps.

**Figure 2:**
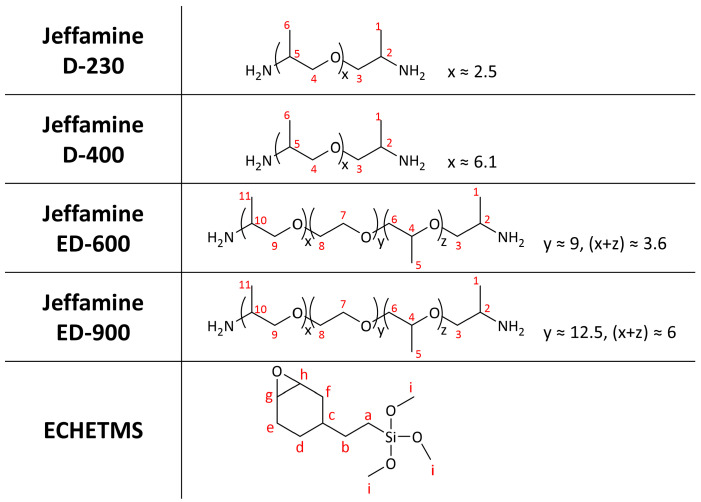
Precursors used on the synthesis of the OIHs.

The four new materials were based on ECHETMS and Jeffamines of different molecular weights (*vide* Figure 2). The hybrids were identified as ACH (Jeff. MW), with ACH standing for the aminociclohexanol group that was formed. A(230) and A(400) matrices are based on D-series Jeffamines D-230 and D-400, respectively, while A(600) and A(900) matrices are based on ED-series Jeffamines ED-600 and ED-900, respectively. The D-series Jeffamines are diamines with a polypropylene glycol (PPG) backbone, while ED-series Jeffamines are diamines with a predominantly polyethylene glycol (PEG) backbone. Carbon atoms were numbered for the sake of simplicity in the analysis.

### 3.2. Nuclear Magnetic Resonance (NMR)

**Figure 3:**
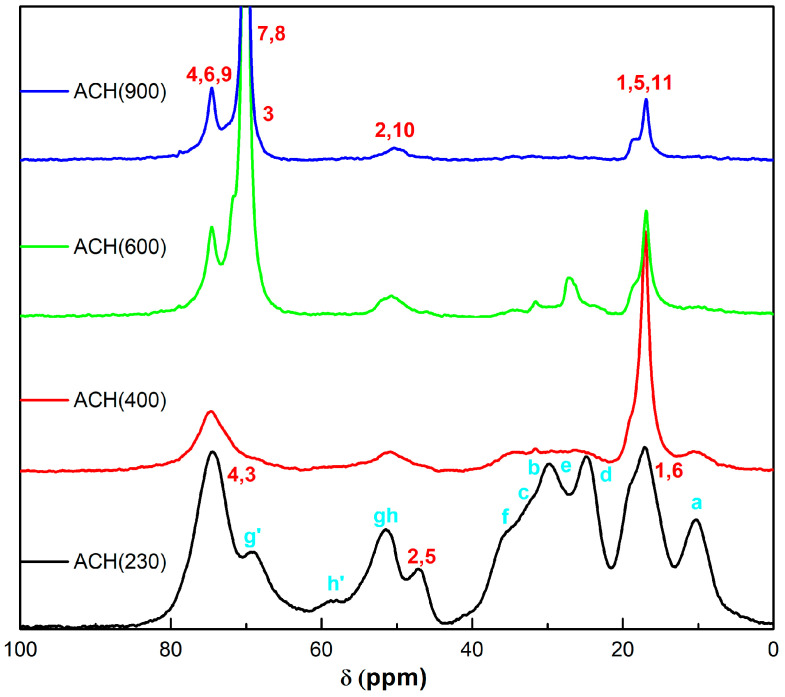
^13^C CPMAS NMR spectra of the new OIH sol-gel matrices.

Solid-state NMR (ssNMR) analysis was performed to structurally characterize the novel matrices. Figure 3 shows the ^13^C CPMAS NMR spectra of sol-gel hybrids. The carbons of the hybrid skeletons are identified in the spectra according to the labelling previously proposed in Figure 2.

Starting from the ACH(230) sample, its spectrum shows the presence of all the signals of the two reagents (Figure S1a,b) with some modifications. In particular, the reaction between Jeffamine and the silane causes the ECHETMS partial ring opening, proved by the presence of two new resonances at 69 and 58 ppm (*g’*, *h’*), with respect to the original *g* and *h* that fall in the broad resonance at about 51 ppm. The *h’* chemical shift, attributable to the C-N bond, together with the shoulder at 35 ppm, attributable to a slight downfield shift of *f*, could support the reaction between the epoxide and the amino groups of the Jeffamine [23]. Moreover, the chemical shift of *a* at 10 ppm and the absence of a sharp peak (methoxy, *i*) at 50 ppm speak for fully hydrolyzed and condensed Si units of the ECHETMS.

These findings can be extended to the other samples through the comparison of the spectra because the higher molecular weights of the Jeffamines hinder a satisfactory discussion of the data. In fact, for sample ACH(400), the signals from the Jeffamine moiety are more significant, due to the higher content of this component. Finally, in the spectra of samples ACH(600) and ACH(900), the hybrids based on ED-type Jeffamines, the signals of the PEG units (7,8) dominate the others, as expected.

**Figure 4:**
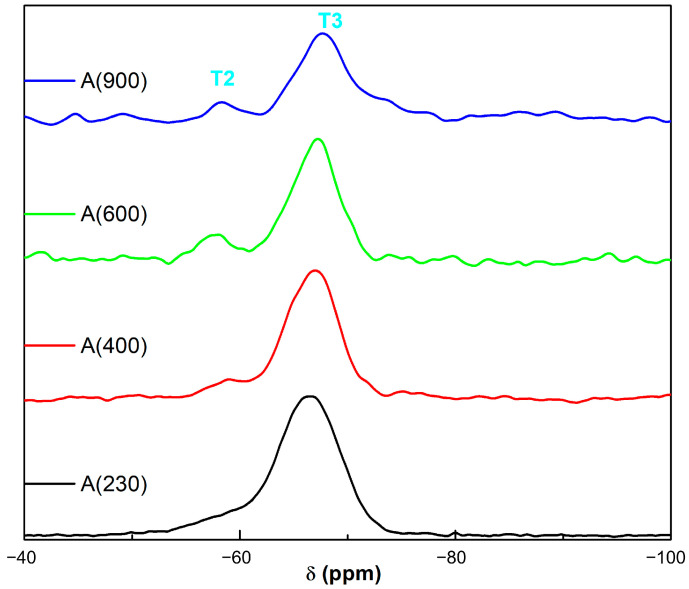
^29^Si MAS NMR spectra of the new OIH sol-gel matrices.

The ^29^Si MAS NMR spectra recorded in OIH samples are shown in Figure 4, where the typical signals of T units (from −50 to −80 ppm) belonging to the trialkoxysilanes present in the different samples are visible (as reported in the Experimental Section: T*^n^* states for trifunctional SiCO_3_ units and *n* the number of siloxane bridges). The results of the profile fitting analysis are reported in Table 1. It appears that these OIH samples present only T^2^ and T^3^ units, whose resonances fall at −58 and −66 ppm, respectively, and a high value of the degree of condensation (DOC), always above 94% (Table 1). This parameter was calculated according to the following equation [24]:

(1) $$\mathrm{DOC}=\frac{\left(2T^{2}+3T^{3}\right)}{3(T^{2}+T^{3})}\times 100$$

### 3.3. Fourier Transform Infrared Spectroscopy (FTIR)

**Figure 5:**
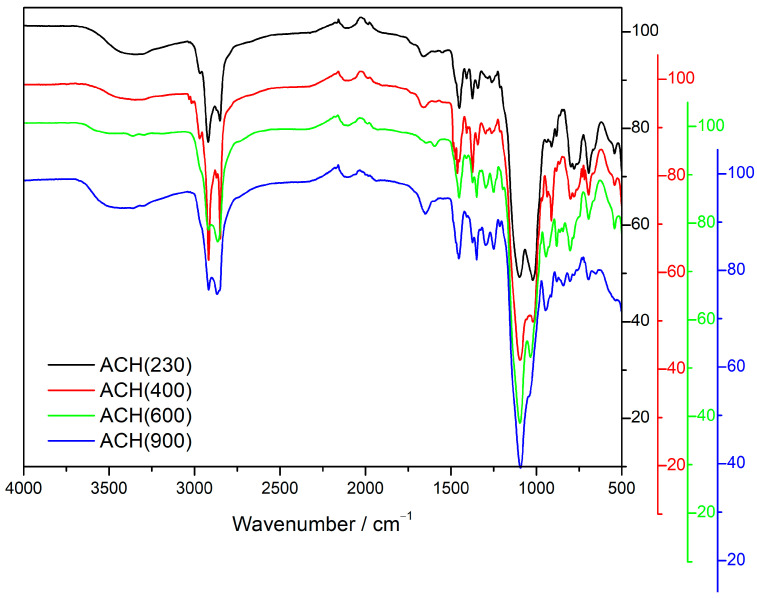
FTIR spectra obtained for the new OIH sol-gel matrices.

Fourier Transform Infrared Spectroscopy (FTIR) was performed for the four new materials to complement the structural characterization performed using ssNMR. Figure 5 shows the FTIR spectra for the four aminoalcohol materials. All four spectra show two strong bands at around 2850–2860 and 2915–2925 cm^−1^, characteristic of C–H stretching vibrations [14,25,26,27,28]. These bands are typical of the organic part from the Jeffamine moiety. The spectra of ACH(230) and ACH(400) show a small peak at around 2970 cm^−1^, which is related to C–CH_3_ asymmetric stretch and is more significant in these two OIHs due to the higher percentage of PPG units. Regarding C–H bending vibrations, the signals can be found at 1450, 1375, and 1350 cm^−1^; the signal at 1375 cm^−1^ can be assigned to the C–CH_3_ bond, which is once again more prominent in A(230) and A(400) hybrids.

The band at 1650–1660 cm^−1^ can be assigned to the C–NH–C bond bending vibration. Together with the absence of typical epoxy signals at 3050 cm^−1^, this confirms the successful reaction between the terminal amine group of Jeffamine moieties and the epoxy group of the silane, as previously indicated by the ssNMR results. The peak at around 1250 cm^−1^ is associated with C–Si bond symmetric bending [29].

The information that the signals in the 1200–1000 cm^−1^ range provide is essential since it is normally independent of the organic moieties linked to silicon [30]. This region can provide important information regarding the architecture of the siloxane skeleton of the material [31]. All spectra show the main peak of the spectra at around 1100 cm^−1^, which can be assigned to Si–O–Si stretching vibration. Another peak at 1035–1020 cm^−1^ appears, with a higher contribution in the OIHs with the lowest Jeffamine MW. According to the literature [29], with longer siloxane chains, the Si–O–Si absorption stretching vibration band becomes broader and more complex. In fact, only cage-type materials show simple Si–O–Si asymmetric stretching vibration bands, while other types of architectural configurations can show complex and ambiguous bands [32]. The stretching vibrations of Si–O–Si bonds show two different symmetric and asymmetric modes. This depends on the parallel or antiparallel displacement of O atoms on the opposite sides of the rings, regarding the inversion through the ring center [31]. This means the higher energy band can be assigned to the asymmetric stretching vibration of Si–O–Si bonds, while the lower energy band regards the symmetric stretching vibration and is not detected in cage-like structures due to the high symmetry of the polyhedral cage. This allows us to conclude that the hybrids with lower MW show less symmetric or random network architectures, while higher MW contributes to a higher symmetry in the hybrid’s architecture.

### 3.4. BET

N_2_ adsorption studies were performed for the four OIH materials. The absorption and desorption isotherm curves are shown in Figure S2. The values obtained from these graphs for surface area (*S*_BET_), pore volume (*V*_pore_), and diameter (*d*_pore_), are summarized in Table 2. Multi-point BET was used to calculate the surface area. The values ranged between 3.3 and 6.3 m^2^/g and do not show any correlation with the MW of the Jeffamine used. V-t method was used to assess the presence of micropores, which were not found in any of the samples. The BJH method found pore volume values corresponding to mesopores and a pattern is shown, with decreasing values with the increase in the organic content of the material. Pore volume was found to be 0.011 cc/g for ACH(230) and 0.007 cc/g for ACH(900). Regarding pore diameter, three of the materials show a bimodal pore distribution; ACH(900) is the material that only shows one pore size. Concerning the smaller value of pore diameter, an increasing value appears with the increase in the MW. Thus, the OIHs with higher organic content, with more symmetric architectures, show higher pore diameter; however, smaller pore volume was observed. Nevertheless, these OIH materials show suitability to be employed as a supporting matrix for doped optical chemosensors, which show generally sizes in the range of a few nm [33].

### 3.5. Optical Analysis

**Figure 6:**
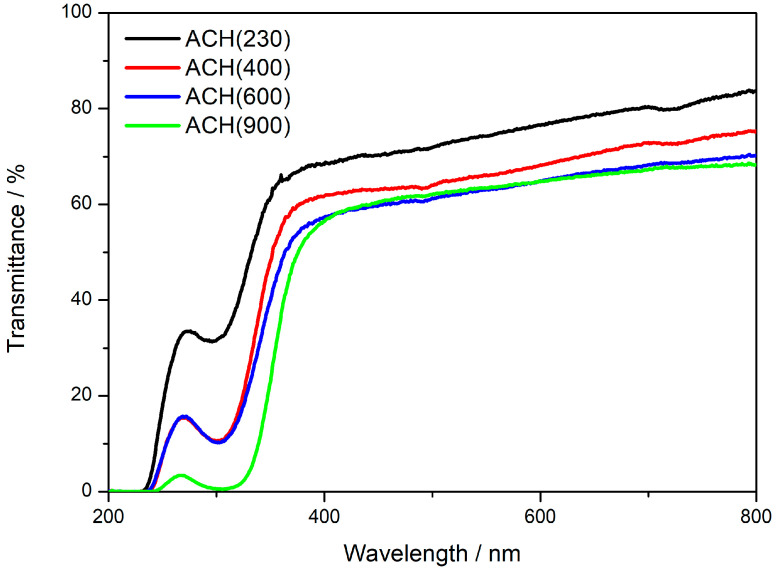
Transmittance spectra of the new OIH sol-gel matrices.

UV/Vis spectroscopy was performed to assess the optical properties of the materials. Figure 6 shows the transmittance spectra for the four new OIH materials. All spectra show a high-transmission region between 350 and 800 nm, as expected for these types of materials [14,15,26]. It is possible to assess that the transparency is higher in the samples with the lower Jeffamine MW, which is according to the information already reported in the literature for OIH materials synthesized with Jeffamine precursors [34]. At 400 nm, the four xerogels show transmittance values of 56%, 57%, 62%, and 68%, for ACH(900), ACH(600), ACH(400), and ACH(230), respectively. The transmittance values slightly increase with higher wavelengths, which is also in agreement with previous reports [9,15,34,35].

The cutoff wavelength (0% transmittance) is around 250 nm for the four materials. However, a peak at around 300 nm is visible in all spectra, being more prominent in the OIH with higher MW. This may be explained by the presence of residual solvents that are entrapped within the crosslinked network [34]. However, it is relevant to mention that the presence of residual solvent within the hybrid structure does not significantly affect the properties of the materials synthesized. Yet, no further conclusions can be drawn. Nevertheless, the obtained results indicate that the OIHs synthesized are suitable for the development of optical sensing materials in the visible zone, by doping optical organic chemosensors that show signal changes in that region.

### 3.6. Electrochemical Impedance Spectroscopy (EIS)

**Figure 7:**
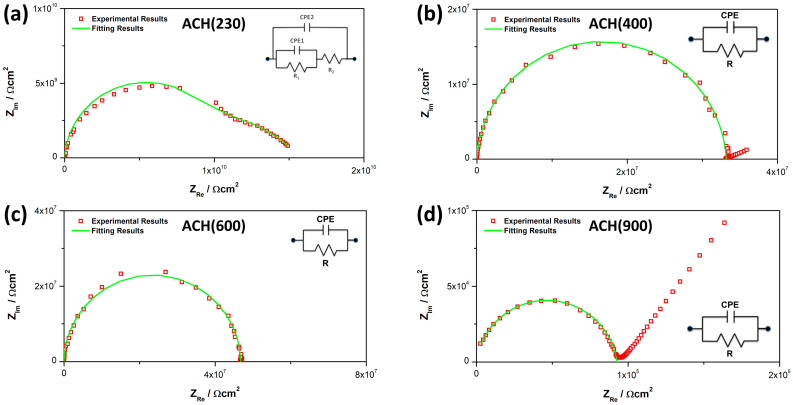
Nyquist plots for the new OIH sol-gel matrices: (**a**) ACH(230); (**b**) ACH(400); (**c**) ACH(600); and (**d**) ACH(900).

Electrochemical Impedance Spectroscopy (EIS) was performed to assess the dielectric constants of the new materials. Nyquist plots illustrate the capacitive response for a broad range of frequencies, and the extracted information (capacitance, conductivity, resistivity, dielectric permittivity, etc.) allows us to quantify the possible degradation of an OIH sol-gel material [36,37,38]. Three measurements were performed in all cases. Figure 7 shows the Nyquist plot for the four OIHs and the equivalent electrical circuit (EEC) is included as inset in each figure. Since in all cases, the results do not show ideal behavior (α ≠ 1), Constant Phase Elements (CPEs) were used instead of pure capacitance.

Regarding ACH(230), the Nyquist Plot show two overlapping semicircles. Therefore, the EEC used to fit the EIS profile contains two CPEs (CPE_1_ and CPE_2_) and two resistances (R_1_ and R_2_). This is related to the two time-dependent charge relaxation processes. The sample’s total resistance (R_sample_) is the sum of R_1_ and R_2_. For the other three samples, the EEC used for the fitting shows only one resistance and one CPE. When the system shows this behavior the impedance of a CPE can be defined as [37]:

(2) $$Z_{CPE}=\frac{R_{sample}}{1+[Q{\left(JW\right)}^{\alpha }R_{sample}]}$$

R_sample_, CPE, and α values, as well as the goodness of the fitting ($\chi^{2}$) are shown on Table 3.

Table 3 shows that the resistance values decrease considerably with the increase in the Jeffamine MW. For ACH(230), the sum of the two R values yields a R_sample_ of 3.22 × 10^10^ Ω cm^2^, while for ACH(900), the value is 9.27 × 10^4^ Ω cm^2^. CPE values are all in the same magnitude order (10^−12^). The values from Table 3, were used to obtain the effective capacitance (C_eff_) by using the Brug et al. relationship [36,39,40]:

(3) $$C_{eff}={\left[{QR_{sample}}^{1-\alpha }\right]}^{1/\alpha }$$

The resistance (R) and capacitance (C) values were obtained using Equations (4) and (5), respectively, with normalization to cell geometry dimensions:

(4) $$R=R_{sample}\times A_{Au}$$

(5) $$C=\frac{C_{eff}}{A_{Au}}$$

The relative permittivity (ε_r_) was determined using Equation (6):

(6) $$\varepsilon_{r}=(\frac{C_{eff}\times d_{sample}}{\;\varepsilon_{0}})\times A_{Au}$$

The conductivity (σ) was determined using Equation (7):

(7) $$\sigma =\frac{\frac{d_{sample}}{A_{Au}}}{R_{sample}}$$

A_Au_ stands for the area of the gold electrodes used on each side of the material for the analysis and d_sample_ stands for the thickness of each film, and ε_0_ stands for the vacuum permittivity. All values are shown in Table 4.

According to Table 4, the resistance of the materials shows a decrease with the increase in the Jeffamine MW. This behavior is according to the literature when this type of polyetheramine was used in OIH synthesis [13]. Moreover, it is possible to assess that the use of ECHETMS instead of GPTMS does not change this feature. C values are all in the same magnitude order (pF cm^2^). This presents a small difference when compared to GPTMS-based aminoalcohols [13], which showed C values in the order of 10 pF cm^2^. This may indicate that the presence of the cyclic ring from ECHETMS may reduce the capacitance value of the final OIH. Conductivity values (σ) show the same pattern as resistance values, with a big decrease with the increase in the MW, which is also according to the literature [13]. Regarding ε_r_ values, the results are between 2.60 and 8.74 and no clear pattern was found linked to the Jeffamine MW.

### 3.7. Thermogravimetric Analysis

**Figure 8:**
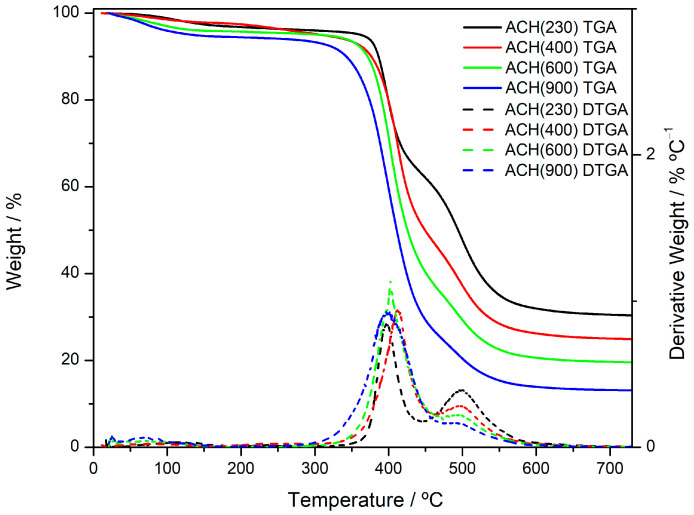
TGA and DTGA traces for the new OIH sol-gel matrices.

Figure 8 shows TGA and DTGA traces for the four OIH matrices. Table 5 shows the 5% weight loss temperature (T_5_), the temperature of the maximum rate of weight loss (T_max_), the activation energy (*E*_A_), and char yield for the four materials, obtained by the TGA and DTGA graphs. Below 150 °C, it is possible to see a small degradation process related to the evaporation of residual water that was still entrapped within the matrix. The weight loss is clearer in ACH(900), which is also confirmed by the T_5_ comparison, which decreases with the increase in the MW and is particularly lower for ACH(900) (134 °C).

The main degradation processes for the four materials occur between 350 and 550 °C. These results are in agreement with the literature, that reports the major degradation processes in amino-alcohol-based OIH materials between 350 and 500 °C [34,41,42,43,44]. Two degradation processes can be seen in the DTGA traces around 400 °C and 500 °C, with different percentages of each depending on the Jeffamine MW. Namely, the second degradation process (at around 500 °C) is more intense for ACH(230) and decreases with the increase in the MW. The first degradation process can be assigned to the depolymerization of the Jeffamine moiety and condensation of residual Si–OH groups [43]. The degradation process around 500 °C can be related to the cleavage of Si–C bonds and to the total oxidation of the organic components, that yields silica/carbon composites. Due to the higher organic content in the material, the OIHs with higher Jeffamine MW show a higher contribution of the first degradation process. This is also correlated by Char Yield values, which are associated with the remaining silica composites and are higher in the lower MW hybrids due to the higher inorganic content. Activation energy (*E*_A_) values were calculated through the Arrhenius equation [45]. The results show high *E*_A_ values for the first degradation process (all above 125 kJ/mol), particularly for ACH(230), which showed an *E*_A_ value of 419 kJ/mol for this degradation process. There are no significant changes on the T_max_ values between the four materials. Besides, below 300/350 °C the materials can be considered thermally stable.

## 4. Conclusions

In this work, four new OIH xerogels, produced via the sol-gel route, are reported for the first time. These materials were successfully synthesized using the precursors silane ECHETMS and the Jeffamines^®^ D-230, D-400, ED-600, and ED-900. The materials were structurally characterized using NMR and FTIR, which confirmed the successful reactions between the precursors and the condensation of the alkoxy groups, with DOC values above 94%. Several techniques were used to assess the suitability of these new OIHs as a supporting matrix for doped chemosensors. BET allows the conclusion that the size pore range of the synthesized OIHs matrices is suitable to be doped with organic chemosensors. The UV/Vis spectroscopy showed that the reported OIHs are suitable for the development of optical sensing materials in the visible zone. The dielectric properties of the OIH materials were assessed using EIS, and these seem to be promising for sensing applications. However, further studies need to be conducted according to the environment chosen. Thermal degradation was characterized using TGA, which allows the conclusion that these materials can only be applied in a temperature ranging from 20 °C to 350 °C. In the end, it can be concluded that these materials showed promising properties to be used as a supporting matrix for doped chemosensors in a wide range of applications.

## Figures and Tables

**Table 1:** ^29^Si MAS NMR: chemical shifts, assignments, and relative amounts of silicon species (all values are reported with a 95% confidence level).

OIH Sample	T^2^ (%)	T^3^ (%)	DOC
*δ* (ppm)	−58.3	−66.3	
ACH(230)	15.4	84.6	94.9
ACH(400)	15.0	85.0	95.0
ACH(600)	14.6	85.4	95.1
ACH(900)	12.9	87.1	95.7

**Table 2:** BET results: surface area *S*_BET_, pore volume *V*_pore_, and diameter *d*_pore_.

OIH Sample	*S*_BET_ (m^2^ g^−1^)	*V*_pore_ (cc g^−1^)	*d*_pore_ (nm)
ACH(230)	6.138	0.011	3.9
ACH(400)	4.572	0.009	3.9
ACH(600)	6.286	0.007	3.9
ACH(900)	3.342	0.007	4.4

**Table 3:** Fitting parameters obtained for the different OIH sol-gel materials.

OIH Films	Rsample/Ω cm^2^	CPE (Q)/S Ω^−1^ cm^−2^	α	$\chi^{2}$
ACH(230)	$R1:\;1.43\;\times \;10^{10}\;(\pm 1.42\%)$ $R2:\;1.79\;\times \;10^{10}\;(\pm 3.46\%)$	$\mathrm{CPE}1:\;2.83\;\times \;10^{-12}\;(\pm 9.23\%)$ $\mathrm{CPE}2:\;1.50\;\times \;10^{-12}\;(\pm 8.71\%)$	α1: 0.969 α2: 0.960	$4.02\;\times \;10^{-3}$
ACH(400)	$3.32\;\times {\;10}^{7\;}\left(\pm 0.52\%\right)$	$1.35\;\times {\;10}^{-12}\;(\pm 1.90\%)$	0.965	$4.18\;\times \;10^{-4}$
ACH(600)	$4.67\;\times {\;10}^{7}\;(\pm 0.51\%)$	$4.40\;\times \;10^{-12}\;(\pm 2.16\%)$	0.988	$6.67\;\times {\;10}^{-4}$
ACH(900)	$9.27\;\times \;10^{4}\;(\pm 0.62\%)$	$4.46\;\times {\;10}^{-12}\;(\pm 7.26\%)$	0.918	$8.26\;\times {\;10}^{-5}$

**Table 4:** Electrical and dielectric properties of the OIH samples for the new OIH.

OIH Sample	log R/Ω cm^2^	C/pF cm^2^	εr	−log σ/S cm^−1^
ACH(230)	10.40 ± 0.07	5.15 ± 0.06	8.74 ± 0.10	11.23 ± 0.04
ACH(400)	7.45 ± 0.03	1.31 ± 0.18	2.60 ± 0.35	8.21 ± 0.06
ACH(600)	7.52 ± 0.30	4.99 ± 0.64	6.09 ± 0.79	8.48 ± 0.37
ACH(900)	4.86 ± 0.04	2.08 ± 0.81	2.85 ± 1.11	5.78 ± 0.12

**Table 5:** Five percent weight loss temperature (T_5_), the temperature of the maximum rate of weight loss (T_max_), and char yield for the OIH films (data obtained from the TGA and DTGA traces).

OIH Sample	T5 (°C)	Tmax (°C)	*E*_A_ (kJ mol^−1^)	Char Yield (%)
ACH(230)	362	397/497	419/100	30.0
ACH(400)	308	413/501	169/43	24.6
ACH(600)	307	403/493	198/18	19.3
ACH(900)	134	397/482	125/2	12.9

## Data Availability

Data sharing is not applicable to this article.

## Supplementary Materials

The following supporting information can be downloaded at: https://www.mdpi.com/article/10.3390/nano13172429/s1, Figure S1: ^13^C NMR spectra of the precursors in CDCl_3_ (whose resonance is a triplet at 77 ppm). (a) ECHETMS (b) D-230 (c) D-400 (d) ED-600 (e) ED-900; Figure S2: N2 adsorption and desorption isotherm curves for the four OIH materials: (a) ACH(230); (b) ACH(400); (c) ACH(600); (d) ACH(900).
