# Supplementary material for: Amino-Alcohol Organic-Inorganic Hybrid Sol-Gel Materials Based on an Epoxy Bicyclic Silane: Synthesis and Characterization
Source: Nanomaterials (Basel). 2023 Aug 26;13(17):2429. doi: 10.3390/nano13172429 (PMC10490488; doi:10.3390/nano13172429)
Supplement: Supplementary file 1 [file nanomaterials-13-02429-s001.zip › nanomaterials-2556401-supplementary.pdf]

nanomaterials

MDPI

Supplementary Materials

Amino-Alcohol Organic-Inorganic Hybrid Sol-Gel Materials Based on an Epoxy Bicyclic Silane: Synthesis and Characterization †

Rui P. C. L. Sousa [1], Rita B. Figueira [1,*], Emanuela Callone [2], Sandra Dirè [2], Susana P. G. Costa [1] and Maria Manuela M. Raposo [1,*]

[1] Centre of Chemistry, University of Minho, Campus of Gualtar, 4710-057 Braga, Portugal; rui.sousa@quimica.uminho.pt (R.P.C.L.S.); spc@quimica.uminho.pt (S.P.G.C.)
[2] "Klaus Müller" NMR Laboratory, Department of Industrial Engineering, University of Trento, Via Sommarive, 9, 38123 Trento, Italy; emanuela.callone@unitn.it (E.C.); sandra.dire@unitn.it (S.D.)
* Correspondence: rita@figueira.pt (R.B.F.); mfox@quimica.uminho.pt (M.M.M.R.)

† This paper is dedicated to the memory of Professor Carlos J. R. Silva who passed away suddenly on 27 August 2020.

a].

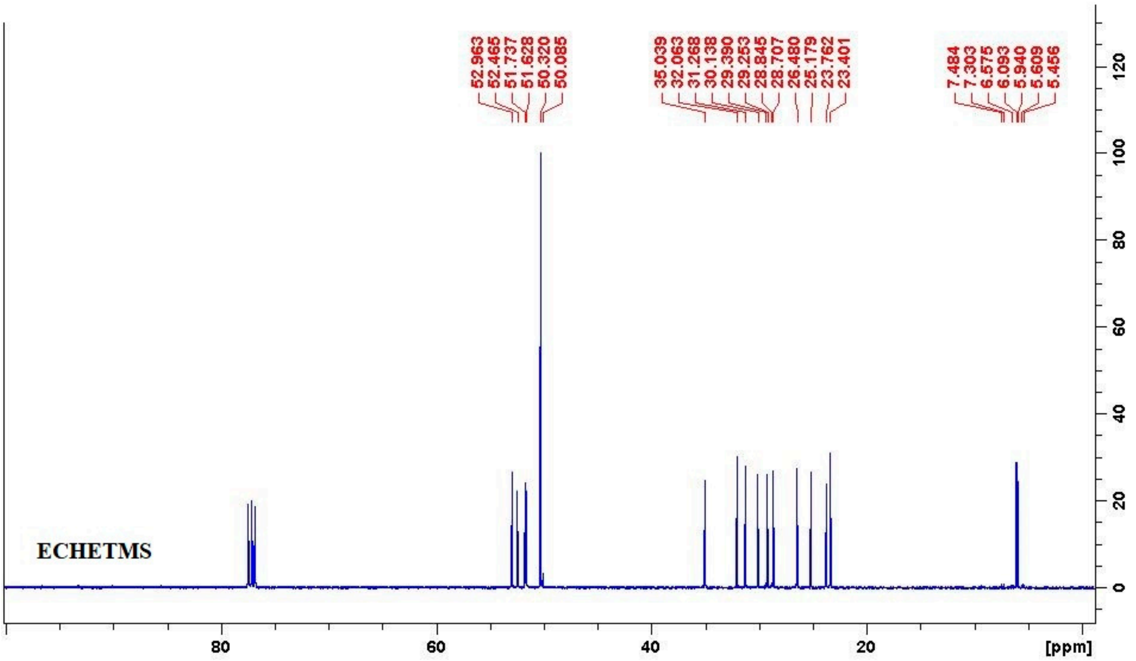

b].

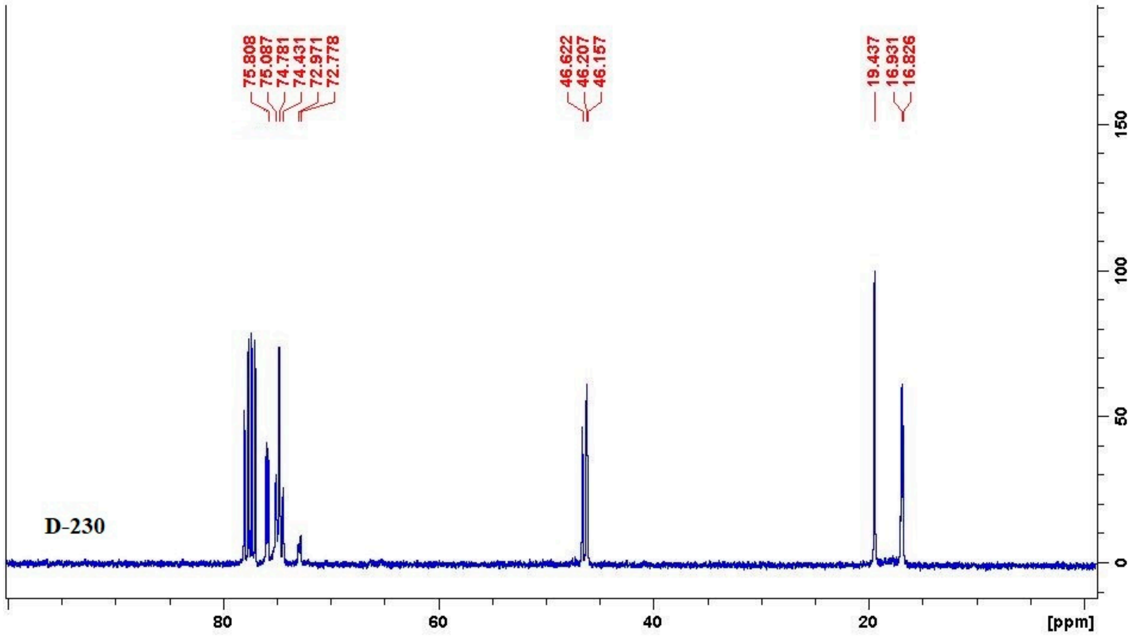
75.808
75.087
74.781
74.431
72.971
72.778
46.622
46.207
46.157
19.437
16.931
16.826
D-230
80
60
40
20
[ppm]
0
50
100
150

c].

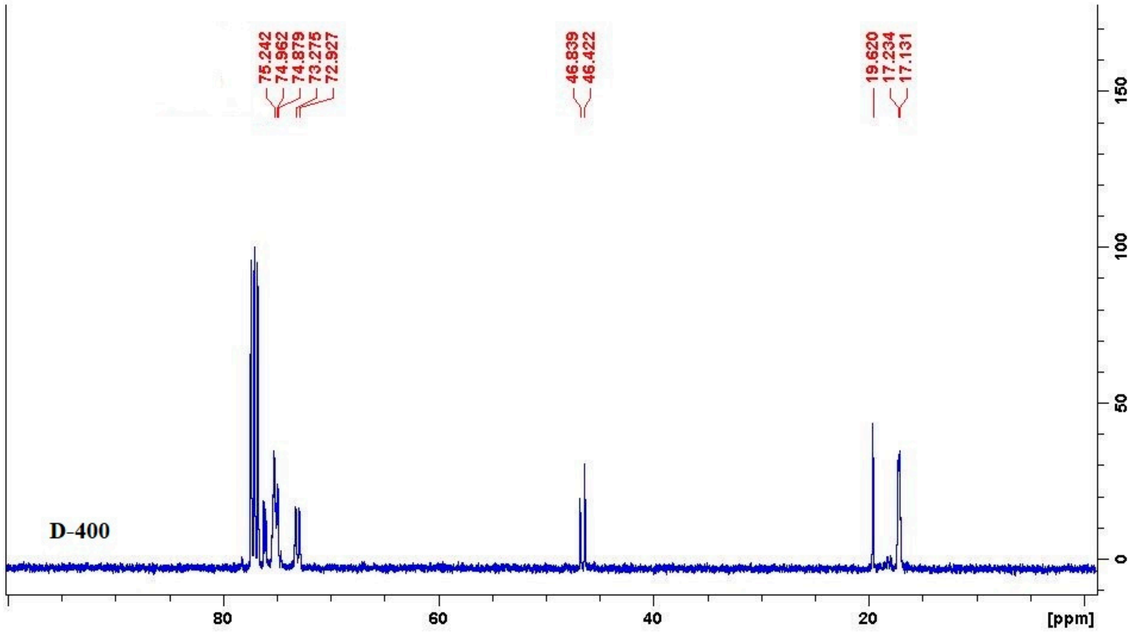
75.242
74.962
74.879
73.275
72.927
46.839
46.422
19.620
17.234
17.131
D-400
80
60
40
20
[ppm]
0
50
100
150

d].

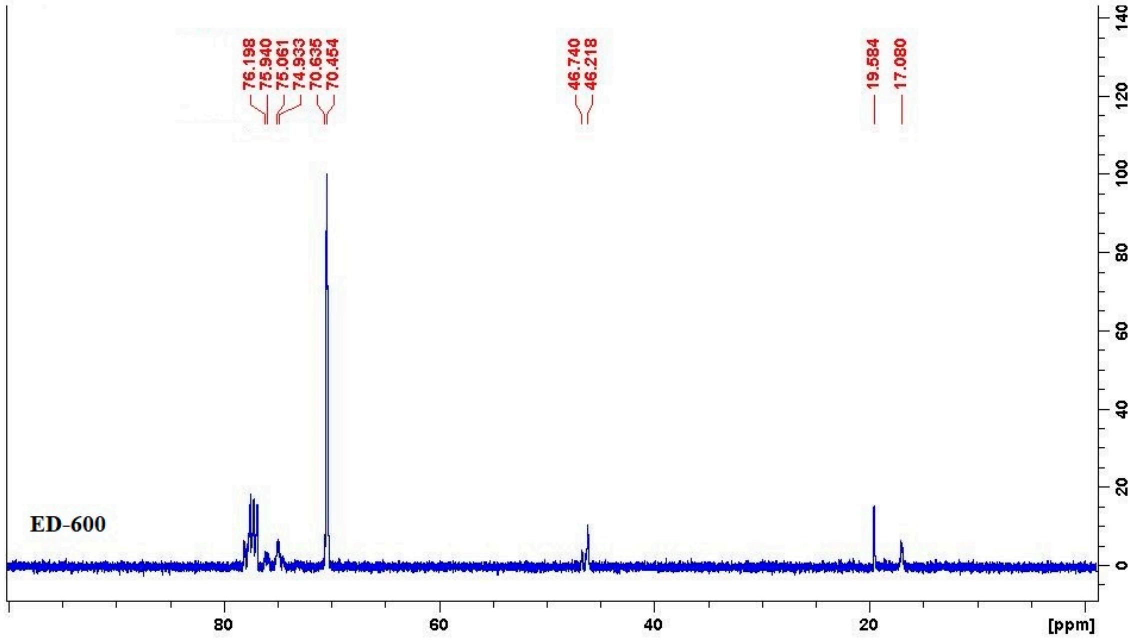

e].

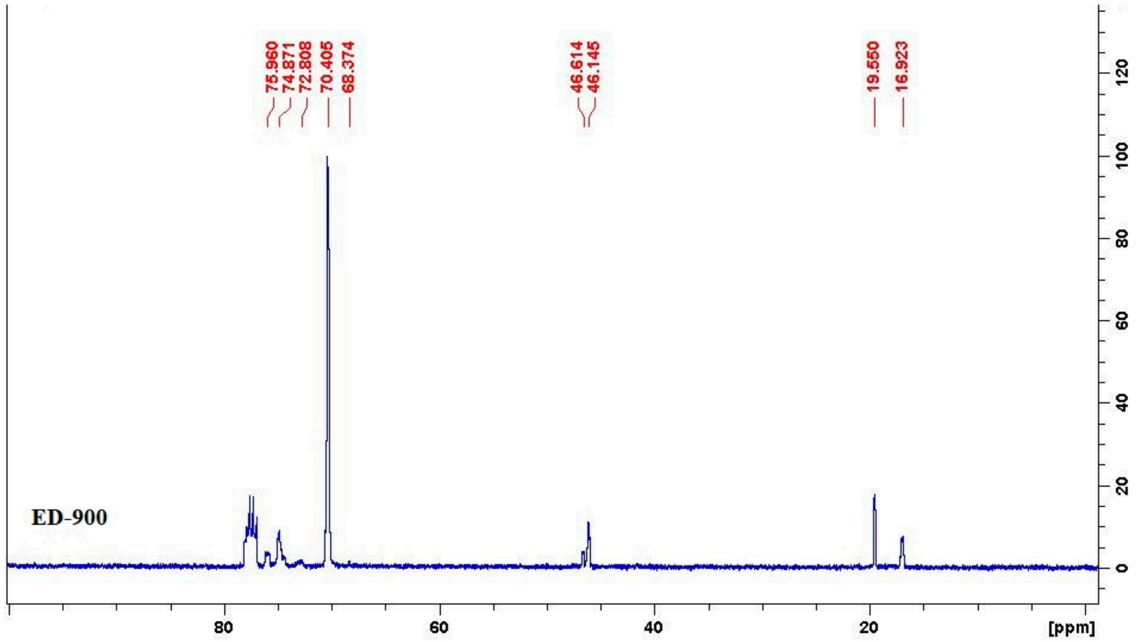

Figure S1: ^{13}C NMR spectra of the precursors in $CDCl_3$ (whose resonance is a triplet at 77 ppm) a) ECHETMS b) D-230 c) D-400 d) ED-600 e) ED-900.

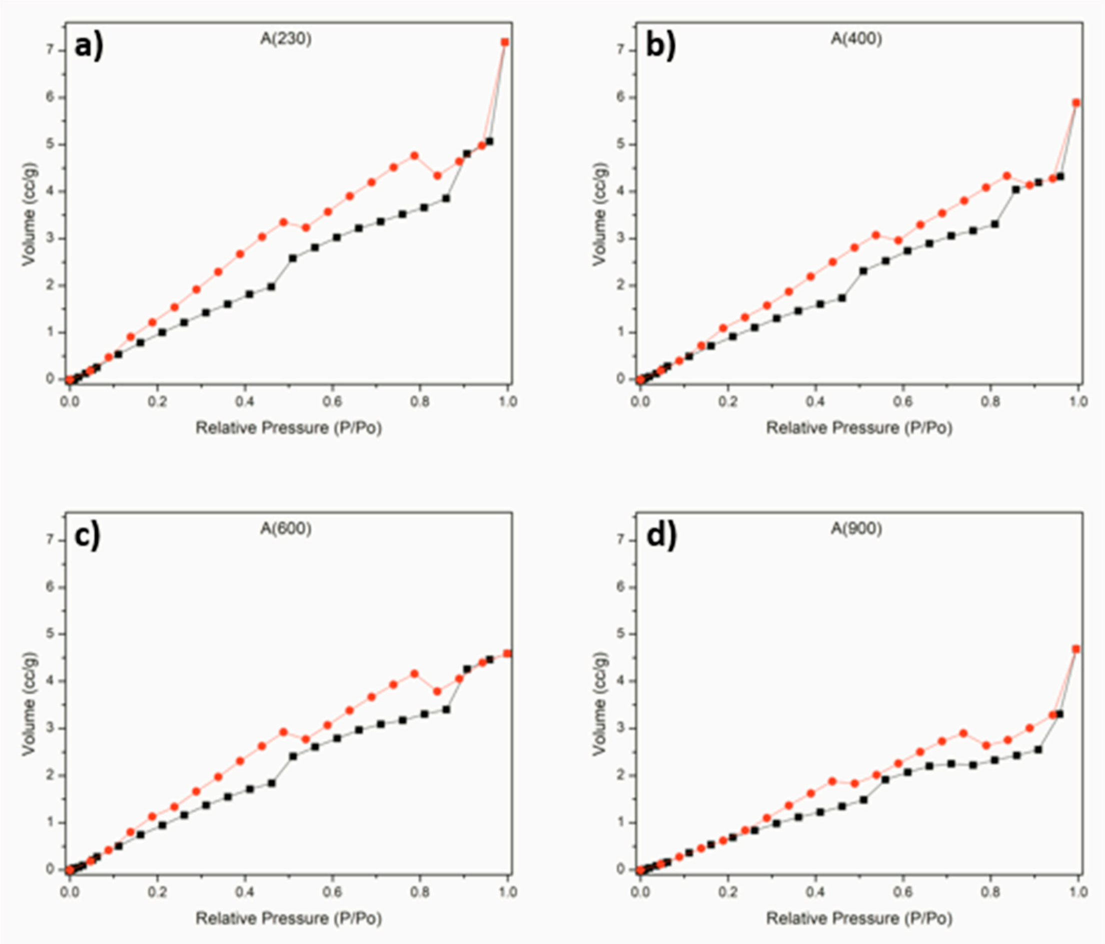

Figure S2. N_2 adsorption and desorption isotherm curves for the four OIH materials: **a)** ACH(230); **b)** ACH(400); **c)** ACH(600); **d)** ACH(900).
